# Editorial: Lysosomal peptidases in tumor immunity

**DOI:** 10.3389/fimmu.2023.1176736

**Published:** 2023-03-14

**Authors:** Milica Perišić Nanut, Biljana Božić Nedeljković, Anahid Jewett

**Affiliations:** ^1^ Department of Biotechnology, Jožef Stefan Institute, Ljubljana, Slovenia; ^2^ Institute for Physiology and Biochemistry “Ivan Djaja” Faculty of Biology, University of Belgrade, Belgrade, Serbia; ^3^ Division of Oral Biology and Medicine, The Jane and Jerry Weintraub Center for Reconstructive Biotechnology, University of California School of Dentistry, Los Angeles, CA, United States

**Keywords:** lysosomal peptidases, immunotherapies, T cell immunity, immunopeptidomics, peptidase inhibitor

The Research Topic “*Lysosomal peptidases in tumor immunity*” aimed to focus on basic and translational research to highlight the role of peptidases in tumor development and to evaluate their potential in cancer diagnosis and therapy. Due to such diverse functions of lysosomal peptidases, it is imperative to understand and delineate the mechanisms by which they regulate immune function in health and disease. Lysosomal peptidases are involved in multiple stages in tumor development and progression. Apart from tumor cells the origin of damaging proteolytic activity can be other cells in tumor microenvironment, such as immune cells that further contribute to tumor progression. Because of their direct relationship in activating cytotoxic granules, lysosomal peptidases can control the cytotoxic functions of both NK and CD8+ T cells. Regulation of cytokines and chemokines by the lysosomal peptidases are paramount to the survival, activation and differentiation of immune as well as tumor cells, rendering immune cells more active and tumor cells less capable of invasion and metastasis. Thus, if any of the functions of such peptidases become compromised, cancer cells will survive and invade. Only comprehensive understanding of pathological mechanisms enables a design of new therapeutic tools directed to harmful proteolytic activity in tumours, without affecting at the same time proteolytic functions involved in antitumor immune responses or other physiological processes. The biogenesis of peptide antigens is a complex process shaped by environmental stimuli and specific proteolytic enzymes located in distinct organelles of the endosomal-lysosomal system. In this collection of articles, new horizons in identification, characterization and modulation of peptide mediated T cell activation has been presented as novel tools are emerging from such studies. Loss of function or downmodulation of lysosomal peptidases in cancer may make such cells invisible to the T cell immune function, however, there are other cells that see lack of MHC-class I, such as NK cells. However, lysosomal peptidases play a significant role in the function of NK cells. By identifying the function of important lysosomal peptidases, we may not only be able to correct and augment their function but also use them as novel biomarkers for targeting tumor cells.

Through their involvement in protein degradation and recycling endosomal-lysosomal peptidases generate vide variety of peptides for presentation *via* MHC class I molecules. Although main immunologically relevant role for HLA class I endo/lysosomal recycling is considered to be in antigen cross-presentation, i.e. the presentation of exogenous antigens *via* MHC class I molecules, endosomal recycling and assembly is also important for constitutive HLA class I induction. Therefore, during infection or cancer, apart from self-proteins, peptides from pathogen or tumour-specific antigens (TSAs) are added to the HLA-I-restricted “ligandome”. However, using conventional methods such as immunoprecipitation/mass spectroscopy identification of HLA-restricted peptides is a complex and time-consuming task. Finton et al., presented ARTEMIS, platform that accurately reports HLA-I ligandomes, regarding the peptides, binding motifs, and length distributions identified. They used this platform to index allele-specific ligandomes from HEK293 cells for several conventional HLA alleles and showed that ARTEMIS can be used to identify new HLA-restricted peptides such as oncoproteins and TSAs. Although it is still based mostly on cell lines due to its requirement for lentiviral transduction, owing to the simplicity of the workflow and its accuracy ARTEMIS represents a powerful tool for identifying potentially clinically useful pHLA targets.

Endosomal-lysosomal cation channels primarily regulate the function of the endolysosomal system trough regulation of osmolarity, pH, membrane potential and trough Ca^2+^ release. However, growing amount of data indicate that channels in the endolysosomal system play a crucial role in the pathology of infectious diseases and cancer. Alharbi and Parrington offered some insights in the role of Ca^2+^ channels in the specific functions of innate and adaptive immune cells. Cation channels including two-pore channel (TPC)1 and TPC2 are particularly important for monocyte function whereas ionotropic nucleotide receptor subunit 4 (P2X4R) is recognized as a key mediator in inflammation. In depth understanding of the mechanism of their actions is of great importance as numerous studies point to these receptors as very promising drug targets for the prevention and treatment of the emerging infectious diseases and cancer.

Immunopeptidomics is a rapidly growing field concerned with the isolation and characterization of MHC-bound peptides, primarily through the use of liquid chromatography and mass spectrometry. Thanks to improvements in instrumentation, the science of nanoscale separation, and data acquisition, remarkably large data sets have recently been obtained. However, it is clear that peptide-centric data analysis pipelines still present informatics challenges, as the proportion of confidently assigned spectra from immunopeptidomic data is significantly lower compared to conventional samples. These issues are particularly evident when it comes to the presence of post-translationally spliced peptides in the immunopeptidome, which were first associated with the immune response in tumors. Nevertheless, their presence has also been found in other diseases, with the abundance of these peptides in the HLA class I immunopeptidome accounting for up to one-third of all peptides. This information has shed light on the very large proportion of peptides that cannot be readily assigned using reference proteomes and has also driven the development of informatics for immunopeptidome data searching. Consensus on this issue has still not been reached, although it is known that between 0 and 45% of peptides are spliced, making it crucial to illuminate this, as stated by Purcell “dark side” of the immunopeptidome.

Tumor-associated antigens (TAA) are cellular self-antigens that are usually overexpressed in tumor cells, but also found at low levels in normal cells, and therefore susceptible to immunological tolerance. Due to this fact, TAA-based tumor vaccines have been shown to have limited efficacy. In order to enhance TAA immunogenicity, Tagliamonte et al. proposed the development of heteroclitic peptides (htcPep) based on prediction algorithms, keeping the same “anchor residues” and mutating the TCR-binding residues only. The main goal is to obtain an epitope sufficiently different from the natural wild-type peptide presented by tumor cells to break immunological tolerance and induce a stronger CD8+ T cell response. Moreover, an MHC-optimized scaffold was designed for enhanced antigen presentation to the TCR by the H2Db allele. This study provides experimental evidence that heteroclitic peptides can be approximated to evoke a stronger T-cell response and the control of tumor growth than the corresponding wild-type peptide, as well as that a universal scaffold for a MHC molecule can be predicted for optimal presentation of different antigen sequences to TCR. Most importantly, this novel approach may be of great clinical importance for tumor vaccine development.

Taken together, these reports emphasize the significant role of recent technological advances and novel database search platforms that have fueled immunopeptidomic discoveries and are opening up new perspectives for cancer immunotherapy.

## Author contributions

All authors listed have made a substantial, direct, and intellectual contribution to the work and approved it for publication.

